# Elastic Stable Intramedullary Nailing (ESIN) in Diaphyseal Fractures of Both Forearm Bones in Children

**DOI:** 10.7759/cureus.106910

**Published:** 2026-04-12

**Authors:** Hafid Talha, Mohammed Tazi Charki, My Abderrahmane Afifi

**Affiliations:** 1 Laboratory of Health Sciences of Errachidia, Faculty of Medicine and Pharmacy of Errachidia, Moulay Ismail University of Meknes, Errachidia, MAR; 2 Department of Pediatric Surgery, Moulay Ali Cherif Regional Hospital Center, Errachidia, MAR; 3 Department of Pediatric Orthopedics and Traumatology, Hassan II University Hospital, Faculty of Medicine, Pharmacy, and Dental Medicine, Sidi Mohamed Ben Abdellah University, Fez, MAR

**Keywords:** diaphyseal forearm fracture, elastic stable intramedullary nailing, esin outcomes, pediatric forearm fractures, pediatric fractures

## Abstract

Background

Diaphyseal fractures of both forearm bones are common in children. Although most cases can be managed conservatively, surgical treatment is indicated for unstable, irreducible, open, or complicated fractures. Elastic stable intramedullary nailing (ESIN) has emerged as a minimally invasive option with favorable functional and radiological outcomes.

Methods

We conducted a retrospective descriptive study including 38 children younger than 15 years with diaphyseal fractures of both forearm bones treated with ESIN in the Department of Pediatric Surgery at Moulay Ali Cherif Regional Hospital, Errachidia, Morocco, between January 2019 and December 2024. Epidemiological, clinical, radiological, therapeutic, and outcome data were collected from medical records and follow-up consultations. Functional outcomes were assessed according to pain, mobility, pronation-supination, and return to daily activities. Anatomical and radiological outcomes were also evaluated.

Results

The series showed a marked male predominance, with 35 boys (92.1%) and 3 girls (7.9%), with a mean age of 13 years. Direct trauma was the main mechanism of injury (n=27, 71.1%), and falls were the leading cause (n=32, 85%). The left side was affected in 24 cases (63.2%). Open fractures were recorded in 10 cases (26.3%), including six stage I (15.8%) and four stage II (10.5%) according to the Cauchoix and Duparc classification. The middle third of the forearm was the most frequent fracture site (n=24, 63.2%), and transverse fractures of both bones were the predominant pattern (n=24, 63.2%). The main indications for ESIN were irreducibility (n=29, 76.3%), instability after orthopedic reduction (n=7, 18.4%), and compartment syndrome (n=2, 5.3%). Open reduction was necessary in five cases (13.2%). Clinically, all patients achieved painless limbs with full strength and return to activities, except for two patients (5.3%) with compartment syndrome who developed persistent neuromuscular sequelae. Radiological outcomes were satisfactory. The main complication was skin irritation related to nail prominence in seven cases (18.4%), without the need for hardware removal.

Conclusion

ESIN is a reliable, minimally invasive, and effective technique for the treatment of pediatric diaphyseal fractures of both forearm bones. It provides high union rates, satisfactory functional recovery, and a low complication profile. ESIN represents a valuable surgical option, particularly for unstable or irreducible fractures.

## Introduction

Diaphyseal forearm fractures in children are common and usually result from various mechanisms, particularly falls from standing height and sports-related injuries [1]. The remodelling potential of diaphyseal fractures is age-dependent, being greater in younger children [2]. Indications for operative treatment of pediatric diaphyseal forearm fractures include unstable fractures, open fractures, failed closed reduction, irreducible fractures, and fractures associated with neurovascular injury [1]. Plate fixation has been reported to provide acceptable results in children. However, it may be associated with important complications, including restricted forearm rotation, neurovascular complications, and, more rarely, radio-ulnar synostosis [3]. Compared with plate fixation, elastic stable intramedullary nailing (ESIN) is associated with less soft tissue dissection, less scarring, and lower rates of nerve injury, infection, and growth arrest [4]. In addition, nail removal is generally considered a simple procedure with a low complication rate [1]. Through this study, we aim to highlight the role of ESIN as a technically simple surgical procedure with good outcomes and a low complication rate, making it a valuable treatment option.

## Materials and methods

Study design and population

We conducted a retrospective descriptive study including 38 children with diaphyseal fractures of both forearm bones treated by ESIN in the Department of Pediatric Surgery at Moulay Ali Cherif Regional Hospital, Errachidia, Morocco, over a six-year period from January 2019 to December 2024.

Inclusion and exclusion criteria

**Figure 1:**
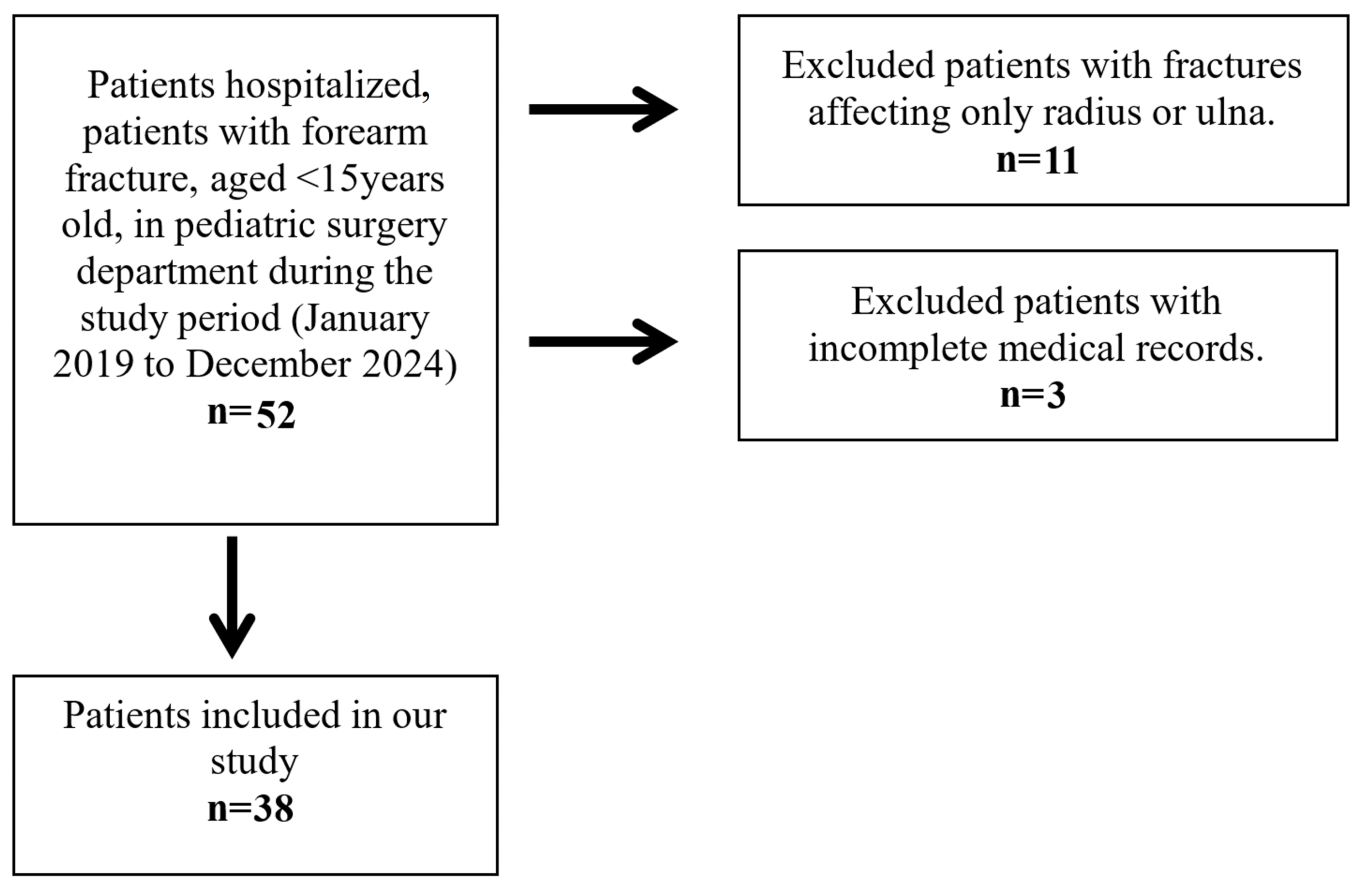
Study flow diagram

The inclusion and exclusion criteria of the study are presented in Table 1. Figure 1 presents the study flow diagram.

**Table 1:** Summary of the exclusion and inclusion criteria of our study ESIN: Elastic stable intramedullary nailing

Category	Criterion
Inclusion	Children younger than 15 years of age
	Diaphyseal fractures of both forearm bones
	Fractures treated with ESIN
	Cases treated with ESIN with or without open reduction of the fracture site
	Recent fractures
Exclusion	Forearm fractures treated orthopedically
	Forearm fractures treated with a surgical technique other than ESIN
	Follow-up of less than 6 months
	Patients lost to follow-up
	Incomplete medical records

Data collection

Between January 2019 and December 2024, we retrospectively reviewed 38 medical records of children with diaphyseal fractures of both forearm bones. Data were collected from patient files and follow-up consultations using a standardized data collection form. The collected variables included epidemiological, clinical, and radiological characteristics, as well as treatment outcomes and postoperative complications.

Data analysis

Data analysis was performed using Google Sheets (Google, Inc., Mountain View, CA), an online spreadsheet tool, to calculate basic descriptive statistics. Diagrams and graphs were also generated using Excel (Microsoft® Corp., Redmond, WA) to provide a clear visualization of the results.

Outcomes were assessed using the global functional forearm score described by Price et al. [5]. This score considers several parameters, including pain, measured using scales adapted for children, joint mobility, particularly elbow and wrist range of motion, muscle strength, pronation-supination, and the ability to resume daily activities.

Functional outcomes were assessed according to the presence or absence of pain at the fracture site, recovery of previous normal activity, and restoration of pronation and supination. Anatomical outcomes were assessed according to scar quality and the presence or absence of residual limb deformity. Radiological outcomes were assessed according to the presence of axial deformity and fracture union.

Results were classified into two categories: satisfactory results, defined as satisfactory findings on all assessed parameters, and unsatisfactory results, defined as impairment on at least one parameter.

Functional score

According to the Price et al. score, outcomes were classified as excellent, good, fair, or poor [5]. Excellent outcomes were defined by mobility of at least 90% of the contralateral healthy side with no pain, whereas good outcomes corresponded to mobility between 75% and 90% of the healthy side with mild pain. Fair outcomes were defined by mobility below 75% of the healthy side with moderate pain, while poor outcomes reflected major functional limitation, severe pain, or the occurrence of complications. This score specifically assessed elbow mobility, wrist mobility, pronation-supination, pain, and the ability to perform daily activities.

## Results

Patient characteristics

A total of 38 children with diaphyseal fractures of both forearm bones were included. There was a marked male predominance, with 35 boys (93%) and 3 girls (7%). The mean age was 13 years, with a range from 6 to 15 years. The most represented age group was 12-13 years, accounting for 22 cases (56%), followed by 10-11 years in 8 cases (22%), while the 6-9-year and 14-15-year groups each accounted for 4 cases (11%). Geographically, most patients came from Errachidia (15 cases, 39%), followed by Tinghir (10 cases, 26%), Midelt (8 cases, 21%), and Figuig (5 cases, 14%). Annual distribution showed fewer cases in 2019 and 2020 compared with later years: four cases in 2019 (10%), three in 2020 (7%), eight in 2021 (22%), seven in 2022 (18%), nine in 2023 (25%), and seven in 2024 (18%).

Mechanism and causes of injury

The mechanism of injury was predominantly direct, observed in 27 cases (71%), whereas indirect trauma accounted for 29% of cases (n=11). Falls were the leading cause overall, representing approximately 85% of etiologies (n=32). More specifically, simple falls were the most frequent cause, reported in 22 cases (58%), followed by falls from height in six cases (17%) and falls on stairs in four cases (10%). Road traffic accidents accounted for four cases (10%), while sports-related accidents were recorded in two cases (5%) (Table 2).

**Table 2:** Summary of fracture mechanisms in our study

Mechanism of fracture	n (%)
Direct trauma	27 (71%)
Indirect trauma	11 (29%)
Falls overall	32 (85%)
Simple falls	22 (58%)
Falls from height	6 (17%)
Falls on stairs	4 (10%)
Road traffic accidents	4 (10%)
Sports-related accidents	2 (5%)

Clinical presentation

The left side was more commonly affected, with 24 cases (63%), whereas the right side was involved in 14 cases (37%). All patients presented to the emergency department with pain and functional impairment of the injured limb. On clinical examination, edema of the traumatized limb was observed in 24 children (63%), and obvious deformity was noted in 12 patients (31%). Two children were admitted after traditional treatment by "Jbira" (based on manual manipulation, followed by improvised external immobilization, often with reeds or simple materials, outside standard orthopedic principles). In addition to the forearm fracture, they presented with blisters, ulcerations, marked limb edema, and decreased finger sensation, requiring operative debridement and excision of the blisters.

Fracture characteristics and associated lesions

**Figure 2:**
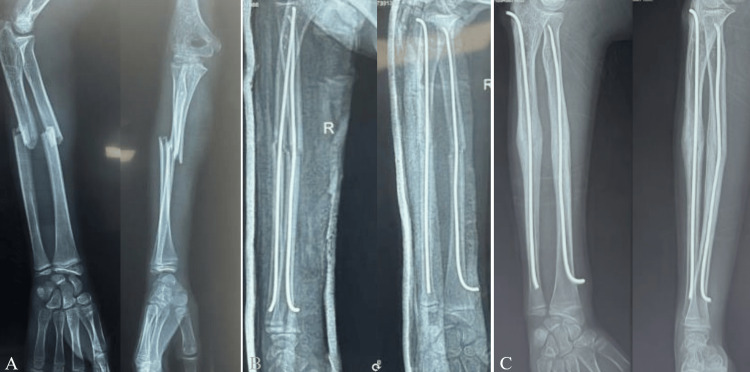
Ten-year-old boy with a displaced overlapping mid-shaft forearm fracture on the preoperative radiograph (A), postoperative radiograph showing intramedullary nails in both forearm bones (B), and radiograph obtained before nail removal demonstrating callus formation with good alignment (C) These images are owned by our patient.

Open fractures were identified in 10 cases, including six classified as Cauchoix and Duparc stages 1 and 4 as stage 2; no stage 3 injuries were reported [6]. No vascular or nerve injuries were found in this series. Most fractures were isolated, representing 37 cases (97%), whereas only one patient (3%) had an associated osteoarticular injury, consisting of a left leg fracture following a road traffic accident. Regarding fracture location, the middle third was the most common site, seen in 24 patients (63%), followed by the distal third in 13 patients (34%), while the proximal third was involved in only one case (3%). Concerning fracture pattern, transverse fractures of both bones were the most frequent type, occurring in 24 cases (63%), followed by oblique fractures of both bones in seven cases (18%), transverse fracture of the radius with oblique fracture of the ulna in five cases (13%), and transverse fracture of the ulna with oblique fracture of the radius in two cases (6%). The most common displacement pattern was overriding, observed in 18 cases (46%) (Figure 2A). Combined angulation and overriding was the second most frequent pattern, seen in 10 cases (25%). Isolated angulation was present in five cases (14%), translation in two cases (6%), isolated radial angulation with a non-displaced ulna in one case (3%), and radial overriding with a non-displaced ulna in two cases (6%).

Surgical indications and postoperative course

The main indications for elastic stable intramedullary nailing were irreducibility of the fracture site in 29 cases (76.3%), instability after initial orthopedic reduction in seven cases (18.4%), and compartment syndrome in two cases (5.3%). Open reduction at the fracture site was required in five cases (13.2%). Postoperatively, all patients were immobilized with a brachio-antebrachio-palmar splint for an average of two weeks (Figure 2B, Figure 2C). The mean hospital stay was four days, with a range of two to six days, and was prolonged in patients with compartment syndrome. Patients were followed clinically and radiologically at one week, at the end of the first month, at the second month, and again for implant removal. Nail removal was performed after bone union under general anesthesia, between the fifth and seventh postoperative months.

Clinical and radiological outcomes

Clinically, all operated patients had painless limbs with full strength, allowing return to activities. However, in the two patients (5.2%) who presented with compartment syndrome, fracture union was achieved, but persistent neuromuscular sequelae remained. Radiologically, outcomes were considered satisfactory overall: 34 patients (91%) achieved anatomical reduction with union within the expected time frame. In the remaining four children (9%), a slight residual displacement persisted, but alignment was considered satisfactory, and the deformity subsequently disappeared with remodeling (Table 3).

**Table 3:** Summary of clinical and radiological outcomes among the patients in our study

Clinical and radiological outcomes	n (%)
Painless limb with full strength and return to activities	36 (94.8%)
Persistent neuromuscular sequelae after compartment syndrome	2 (5.2%)
Anatomical reduction with union within the expected time frame	34 (91%)
Slight residual displacement with satisfactory alignment and subsequent remodeling	4 (9%)

Complications

Complications were mainly represented by skin irritation caused by the protruding nails. This was observed in seven cases (20%), with nail extrusion through the entry site, although none of these cases required hardware removal. Two patients required physiotherapy; both had initially presented with compartment syndrome due to "Jbira" use, and their course was marked by persistent neuromuscular sequelae. No other complications were reported, particularly no infection, nonunion, or malunion.

## Discussion

ESIN has been reported to offer several advantages over other surgical techniques, particularly through preservation of the fracture hematoma and periosteum [7]. However, it may also be associated with complications, including pin migration, infection, nerve injury, radio-ulnar synostosis, delayed union, tendon rupture, and secondary displacement [8]. Some late complications, such as osteitis, may also occur, although they remain rare despite preventive measures [9]. Non-union is uncommon. Delayed callus formation may occur in the presence of excessive motion at the fracture site or, conversely, overly rigid fixation [10]. One of the main technical difficulties of ESIN is crossing the fracture site, with a risk of pin deviation into the surrounding soft tissues [11]. In a series of 125 fractures treated with ESIN, Lascombes et al. reported 2 tendon injuries, 3 cases of transient hypoesthesia, 11 cases of skin irritation, 2 cases of pin twisting, 1 case of pin breakage, 1 case of delayed union, and 5 cases of refractures [12].

Epidemiological profile

Our series showed a marked male predominance, with a sex ratio of 11.6. This finding is consistent with the literature, in which boys are more frequently affected than girls, probably because of greater exposure to trauma during physical activity. However, the male predominance in our study was even more pronounced than in other reported series, including those of Andaloussi et al. [10] and Baghdadi [13]. The mean age in our series was 13 years, similar to that reported by Baghdadi [13], whereas most other studies reported a lower mean age, generally ranging from 8.5 to 11.5 years. This may reflect our tendency to indicate surgery in older children, in whom remodelling potential is lower and anatomical reduction becomes more important.

Trauma mechanism, etiology, and side involved

In our study, the predominant mechanism was direct trauma, which differs from several published series reporting a predominance of indirect trauma, including those by Andaloussi et al. [10], Baghdadi [13], and Saleh Khader [14]. Kwas et al. reported several mechanisms, mainly falls from standing height (32%), followed by sport- and recreation-related trauma (29%) [1]. This discrepancy may be related to differences in how the mechanism of injury was described by families in our setting. Regarding etiology, simple falls were the most frequent cause in our series (58%), in agreement with most reports. Saleh Khader also found that more than half of fractures followed a simple fall [14], while Echarri similarly reported falls as the leading cause [15]. In contrast, Acharya et al. found sports accidents to be the main etiology [16]. Left-sided injuries predominated in our series (63%), consistent with most comparative studies, including those of Saleh Khader [14] and Echarri et al. [15]. This predominance may be explained by right-handedness, with the left upper limb acting as a protective limb during falls or aggression.

Fracture characteristics

The middle third of the forearm was the most frequent fracture site in our series (63%), in agreement with Saleh Khader [14] and Baghdadi [13], whereas Echarri et al. [15] reported a predominance of distal-third fractures. Distal fractures are often easier to manage orthopedically, which may partly explain their lower representation in our surgical series. All fractures in our series were displaced, and overriding was the predominant displacement pattern. This was similar to the findings of Baghdadi [13], whereas Echarri et al. [15] and Acharya et al. [16] reported angulation as the most frequent displacement pattern.

Indications for ESIN

Pediatric diaphyseal forearm fractures were historically treated non-operatively with closed reduction and casting [17]. Despite the thick periosteum and strong remodelling potential in children, closed reduction and cast immobilisation may fail at a considerable rate [18], which supports the need for surgical treatment, particularly plate fixation or intramedullary nailing [19]. Shoemaker et al. proposed that the fixation method used in children should ensure good alignment, technical simplicity, and minimal invasiveness [20]. Compared with other techniques, such as plate-screw fixation, intramedullary nailing appears to fulfil these criteria more closely [17]. Common intramedullary devices include Steinmann pins, K-wires, Rush pins, and elastic titanium nails, with titanium increasingly preferred over stainless steel because of its greater elasticity [17].

Orthopedic treatment remains the first-line treatment for pediatric both-bone forearm fractures, and ESIN is mainly indicated when reduction is impossible or when the fracture remains unstable after reduction [21]. In our experience, only 38 of 180 children with both-bone forearm fractures were managed surgically with ESIN, corresponding to 21%. This rate was close to that reported by Echarri et al. [15] at 20%, but clearly lower than the 55% reported by Saleh Khader [14]. In our series, the main indications were irreducibility and post-reduction instability, which were also the major indications in other studies such as Andaloussi et al. [10]. Monteggia fracture-dislocation, described as a special situation requiring stable fixation of the ulna [22], and compartment syndrome were other particular indications. The predominance of mid-diaphyseal fractures in our cohort was considered particularly suitable for ESIN, in agreement with the literature [14].

Single-bone intramedullary nailing for both-bone forearm fractures has been reported to provide outcomes comparable to fixation of both bones, particularly when only the radius is nailed [23], as reported in the series of Alnaib et al., supporting radius-only fixation [19]. This approach ensures stabilization of the radius while allowing the ulna to rotate into reduction [23]. Alnaib et al. reported a 100% union rate, with no cases of infection, paraesthesia, nail removal complications, or persistent loss of motion, demonstrating excellent functional outcomes, reliable union, and no treatment failures [19].

Role of age in surgical decision-making

Age was considered an important factor in deciding on surgical treatment. In our study, children younger than six years were considered more suitable for orthopedic treatment because residual deformity may still remodel with growth. This view is supported by other series [14]. By contrast, in older children and adolescents, surgical treatment becomes more justified for unstable or irreducible fractures because remodelling potential decreases and residual deformities may compromise forearm rotation. Angulation greater than 15°, narrowing of the interosseous space, overlap, and rotational abnormalities make surgical fixation more necessary in this age group [24].

Open reduction and technical considerations

Open reduction was required in five cases (13%) in our series because closed reduction and nail passage were impossible. This rate was lower than in older series such as Chafaqi [25], in which direct exposure was much more frequent, and also lower than that reported by Dendane et al., who found open reduction in 19% of cases due to muscular or periosteal interposition [26]. More recent series, including those of Saleh Khader [14] and Echarri et al. [15], reported direct surgical exposure only rarely, mainly in unstable fractures or when percutaneous reduction failed. In our study, the radius was nailed retrograde from the distal metaphysis and the ulna antegrade from the proximal metaphysis, consistent with the approach described by Dendane et al. [26]. In the series by Rokaya et al., closed reduction was successful in 25 patients, whereas open reduction was required in 30.6% of cases [17].

Postoperative immobilization and implant removal

All patients in our series received a brachio-antebrachio-palmar splint for two weeks, mainly for pain relief and soft-tissue healing. Postoperative immobilization remains debated. Some teams use splinting for two to three weeks, whereas recent publications suggest that ESIN can be performed safely without postoperative casting, with faster recovery and better patient satisfaction. In contrast, Andaloussi et al. used postoperative splint immobilization for a mean of four weeks [10], and Bergerault [27] maintained splinting until consolidation when fixation was considered less stable. In our series, implant removal was generally planned around six months, which was similar to Bergerault [27], whereas Lascombes et al. preferred later removal, around 10 months, to reduce the risk of refracture [28]. Nail ends are often left accessible to facilitate removal [29]. Earlier removal has been associated with refracture in other reports, including those of Lascombes et al. [9] and Bergerault [27].

Complications

The main complication in our series was skin irritation related to the nail ends. Although the nails were routinely buried subcutaneously, cutaneous irritation or skin breach occurred in six to seven cases without requiring hardware removal. This is consistent with the literature, which identifies ulnar skin irritation as the most frequent complication. Andaloussi et al. reported irritation in 17% of cases [10], while Luhmann et al. reported 11 cases among 125 fractures [30]. Careful cutting of the nails, leaving only 3-5 mm outside the bone, improves tolerance and reduces soft-tissue irritation. Osteitis was not observed in our series, whereas Lascombes et al. and Andaloussi et al. reported approximately 2% of cases, particularly in open fractures [9,10]. No loss of reduction, pseudarthrosis, refracture, or sepsis occurred in our series. By comparison, Lascombes et al. reported one case of sepsis and three refractures [9], while Andaloussi et al. reported three refractures on retained hardware and one proximal radio-ulnar synostosis [10]. In the series by Rokaya et al., complications affected only eight of 36 patients, and only one patient developed delayed union. Other reported complications include nerve-related injuries, emphasizing the importance of careful exposure of the radial entry site and meticulous soft-tissue dissection [17].

Final outcomes

The final outcomes in our series were considered satisfactory. Clinically, all operated children had painless limbs with full strength and were able to resume activities, except for the patients with compartment syndrome, who retained neuromuscular sequelae despite fracture healing. Radiologically, 91% achieved anatomical reduction and union within the expected time frame, while the remaining cases showed only minor residual displacement that remodelled over time. These findings are in line with the literature, in which satisfactory results are reported in the great majority of cases. Similarly, Hossain et al., in a series of 67 children aged 6-15 years treated with ESIN and assessed using the Price score, reported excellent functional outcomes in 86.56% of patients [31]. Overall, ESIN provides reliable stabilization with good functional and radiological results while preserving the biological advantages of closed treatment and minimizing septic risk. Rokaya et al. reported good or excellent outcomes in 32 of 36 patients and only four fair outcomes according to the Clavien-Dindo classification [17]. Martus et al. reported similar results, with 187 of 200 patients having good outcomes [32].

Study limitations

Our study has several limitations. Its retrospective design may have introduced selection and information bias, as data were collected from medical records with potential inconsistencies or missing information. The relatively small sample size (38 patients) and the single-center setting may limit the generalizability of the findings. In addition, the absence of a control group treated with alternative methods (e.g., conservative treatment or plate fixation) prevents direct comparison of outcomes. Finally, the follow-up duration, although sufficient to assess short- to mid-term outcomes, may not fully capture long-term complications or functional sequelae.

## Conclusions

ESIN represents a safe, minimally invasive, and effective option for the management of pediatric diaphyseal fractures of both forearm bones requiring surgical treatment. In this series, ESIN achieved high union rates, satisfactory radiological alignment, and favorable functional outcomes, with a limited complication profile. Its technical advantages, including a limited soft-tissue dissection and a straightforward implant removal, support its widespread use in current practice. Despite the occurrence of minor complications, mainly related to nail prominence, the overall results were satisfactory. ESIN therefore appears to be a valuable surgical option, particularly in unstable or irreducible fractures. Further prospective comparative studies are needed to confirm these findings and better define the respective role of ESIN among available fixation techniques.
